# Effects of the Olive-Derived Polyphenol Oleuropein on Human Health

**DOI:** 10.3390/ijms151018508

**Published:** 2014-10-14

**Authors:** Barbara Barbaro, Gabriele Toietta, Roberta Maggio, Mario Arciello, Mirko Tarocchi, Andrea Galli, Clara Balsano

**Affiliations:** 1 Laboratory of Molecular Virology and Oncology, Francesco Balsano Foundation, Rome 00198, Italy; E-Mails: barbara.barbaro@fondazioneandreacesalpino.it (B.B.); roberta.maggio@fondazioneandreacesalpino.it (R.M.); mario.arciello@uniroma1.it (M.A.); 2 Department of Experimental Oncology, Regina Elena National Cancer Institute IRCCS, Rome 00144, Italy; E-Mail: toietta@ifo.it; 3 Department of Internal Medicine and Medical Specialties, Sapienza University, Rome 00161, Italy; 4 Department of Experimental and Clinical Biomedical Sciences, University of Florence, Florence 50139, Italy; E-Mails: mirko.tarocchi@unifi.it (M.T.); andrea.galli@unifi.it (A.G.); 5 Institute of Biology, Molecular Medicine and Nanobiotechnologies (IBMN), National Research Council (CNR), Rome 00185, Italy

**Keywords:** olive, oleuropein, Mediterranean diet, polyphenols, antioxidant, anti-inflammatory, anticancer, non-alcoholic fatty liver disease

## Abstract

The use of the products derived from the olive tree on human health dates back centuries. In several civilizations, the olive tree had and still has a very strong cultural and religious symbolism. Notably, the official seal and emblem of the World Health Organization features the rod of Asclepius over a world map surrounded by olive tree branches, chosen as a symbol of peace and health. Recently, accumulating experimental, clinical and epidemiological data have provided support to the traditional beliefs of the beneficial effect provided by olive derivates. In particular, the polyphenols present in olive leaves, olives, virgin (unrefined) olive oil and olive mill waste are potent antioxidant and radical scavengers with anti-tumor and anti-inflammatory properties. Here, we review the positive impact on human health of oleuropein, the most prevalent polyphenol present in olives. In addition, we provide data collected in our laboratory on the role of oleuropein in counteracting lipid accumulation in a mouse model of non-alcoholic fatty liver disease.

## 1. Introduction

Archeological evidence suggests that Neolithic inhabitants of the Mediterranean basin have collected and consumed olives since the copper age (sixth millennium BC) and that during the third millennium BC, the cultivation of olive trees and oil production were well established in the region. Over the centuries, olive oil has been used as a cosmetic and pharmacological agent [1]. Recently the beneficial effects of virgin olive oil have been ascribed to the content of polyphenols, which exert antioxidant, anti-inflammatory, anti-cancer, antimicrobial, antiviral, anti-atherogenic, hypoglycemic, hepatic-, cardiac- and neuro-protective effects [2,3,4]. Virgin olive oil is consumed unrefined, and humans absorb a large part of the ingested olive oil phenols [5]. Oleuropein, the molecule responsible for unprocessed olives characteristic bitter taste [6], is the most prevalent phenolic component in olive leaves, seed, pulp and peel of unripe olives (up to 14% of the dry weight) (Table 1) [1]; during fruit maturation, oleuropein undergoes hydrolysis, yielding different products, including hydroxytyrosol (2-(3,4-dihydroxyphenyl)ethanol). Olive variety and the process used for making olives edible highly affect oleuropein content in table olives [7]. Similarly, several factors, including the kind of olive fruit, the ripening stage, the oil production and extraction technologies, determine the final content of oleuropein in virgin olive oil [8]. In addition, the difference in the methods used for oleuropein analysis may account for the variability of the reported oleuropein content in several sources (Table 1). In 1959, oleuropein was isolated and its chemical structure defined [9], opening the way to a more precise understanding of the molecular basis of its action.

Here, we review major clinical and experimental evidence underlining the pharmacological properties of oleuropein on human health. In addition, we provide original data on the role of oleuropein in counteracting lipid accumulation in a mouse model of non-alcoholic fatty liver disease (NAFLD).

**Table 1:** Oleuropein content range in different sources.

Source	Oleuropein Content	References
Olive leaves	93–134 mg/g (DW)	[10]
	6.1–13.3 mg/g (DW)	[11]
	5.6–9.2 mg/g (DW)	[12]
	34.0–38.1 mg/g (FW)	[13]
	60–90 mg/g (DW)	[14]
	2.1–24.8 mg/g (DW)	[7]
Olive branches	11–14 g/kg (DW)	[15]
	18.9 g/kg (DW)	[16]
Olive roots	1.9–6.0 g/kg (DW)	[17]
Olive buds	15.7–58.4 mg/g (FW)	[13]
Olive flowers	15.3–20.9 mg/g (FW)	[13]
Olives (fruit)	2.5–8.9 mg/g (FW)	[18]
	0.6–1.1 mg/g (DW)	[12]
	13.6–50.8 mg/g (FW)	[13]
	0.4–21.7 mg/g (DW)	[7]
	1.3–5.8 mg/g (FW)	[19]
	0.3–3.5 mg/g (FW)	[20]
Table olives	0.0–0.1 mg/g (DW)	[7]
	0.0–0.5 mg/g (FW)	[21]
Virgin olive oil	0.0–11.2 mg/kg	[22]
	0.0–4.7 mg/kg	[23]
	2.0 mg/kg	[24]
	3.8 mg/kg	[25]
Olive oil	Virtually absent	[26]
Olive pomace	0.4 mg/g (DW)	[27]
Olive mill waste water	6.5 mg/g (DW)	[8]
	Absent	[28]

FW: fresh weight; DW: dry weight.

## 2. Effects of Oleuropein on Human Health

### 2.1. Antioxidant Effect

The antioxidant effect of oleuropein is exerted through different mechanisms, resulting in an enhancement of the antioxidant response [2,29]. Oleuropein’s antioxidant potential is mainly related to its ability to improve radical stability through the formation of an intramolecular hydrogen bond between the free hydrogen of the hydroxyl group and its phenoxyl radicals [30].

Oleuropein may counteract oxidative stress, as assessed *in vitro* by the 2,2-diphenyl-1-picrylhydrazyl radical (DPPH) test, demonstrating an antioxidant potential similar to those exerted by ascorbic acid (vitamin C) and α-tocopherol (vitamin E) [30]. Oleuropein has a protective effect in counteracting low-density lipoprotein (LDL) oxidation, both *in vitro*, inhibiting, in a dose-dependent manner, LDL copper-induced oxidation [31,32], and *in vivo*, reducing plasmatic levels of total, free and ester cholesterol in rabbits [33]. The protective effects of oleuropein on lipid oxidation was demonstrated through the evaluation of the decreased formation of thiobarbituric acid-reacting substances (TBARS) and of lipid peroxides by-products, such as malondialdehyde (MDA) and 4-hydroxynonenal (4-HNE) [32]. Moreover, Visioli *et al.* demonstrated in healthy volunteers that administration of oleuropein decreases, in a dose-dependent manner, the urinary excretion of 8-iso-PGF2α, indicating lower *in vivo* lipid peroxidation [34]. A scavenging effect of oleuropein was also demonstrated with respect to hypochlorous acid [30], a potent oxidant species produced *in vivo* by neutrophils myeloperoxidase at the site of inflammation [35]. Oleuropein has also the ability to scavenge nitric oxide (NO); in addition, it also promotes the expression of the inducible nitric oxide synthase (iNOS) in cells [36].

Beneficial effects of oleuropein were also described with regard to heart damage, as demonstrated in isolated rat hearts subjected to 30 min of no-flow global ischemia and then reperfused [37]. Data evidenced a reduction of creatine kinase and reduced glutathione release in the perfusate. Either the oxidized glutathione and the extent of lipid peroxidation were reduced, strengthening the hypothesis that oleuropein may exert antioxidant beneficial effects in the prevention of coronary heart disease [37].

### 2.2. Anti-Inflammatory and Anti-Atherogenic Effects

Some studies documented that oleuropein elicits anti-inflammatory effects by lipoxygenase activity, production of leukotriene B4 [38], inhibiting biosynthesis of pro-inflammatory cytokines [39] or modulating inflammatory parameters [40]. In particular, Impellizzeri *et al.* [40] reported that administration of oleuropein in a mouse model of carrageenan-induced pleurisy causes a significant reduction of tumor necrosis factor α (TNF-α), interleukin-1 beta (IL-1β) and nitric oxide (NO).

The inflammatory response involves non-cellular and cellular components. Potent pro-inflammatory cytokines include TNF-α and IL-1β, which are synthesized immediately after injury. TNF-α and IL-1β are involved in a wide range of events, including vascular permeability [41], recruitment of inflammatory cells [42], induction of inducible iNOS and cyclooxigenase-2 (COX-2) at the injury site. iNOs is one of the three distinct enzymes that produce NO, a free radical gas molecule, which has a crucial role in the development of the secondary inflammatory response. Similar to iNOS, the expression of COX-2, an enzyme involved in the generation of some inflammatory mediators, is also mediated by TNF-α and IL-1β. Khalatbary *et al.* demonstrated that oleuropein treatment significantly attenuates the expression of TNF-α and IL-1β, and, consequently, the expression of iNOS and COX-2 [43].

Olive-derived phenolic compounds, including oleuropein, can decrease the production of monocytic inflammatory mediators, decreasing the production of IL-1β in human whole blood cultures stimulated with monocytes-triggered by LPS [44]. Interestingly, olive oil phenolic compounds decrease the circulating concentrations of IL-6, a pro-inflammatory agent that stimulates inflammation in several pathologies [45]. As reported by Puel *et al.* [46], oleuropein is able to elicit protective effects on bone loss in a model of ovariectomy associated with inflammation, probably by modulating parameters of inflammation (such as fibrinogen and spleen weight).

Oleuropein is endowed also with antithrombotic and anti-atherogenic properties, which, at least in part, depend on its anti-inflammatory and anti-oxidative activities. The protective effect of oleuropein against post-ischemic oxidative burst was investigated by measuring the release in the coronary effluent of oxidized glutathione, a sensitive marker of the heart’s exposure to oxidative stress [37]. Reflow in ischemic hearts is accompanied by a prompt release of oxidized glutathione; in ischemic hearts pretreated with oleuropein, this release was significantly reduced. Moreover, oleuropein has an additional beneficial effect on several aspects of cardiovascular disease via its vasodilatory, anti-platelet aggregation, anti-ischemic and hypotensive properties [47,48,49]. Furthermore, olive-derived polyphenols, including oleuropein, promoting generation of NO from nitrite in the stomach, induce smooth muscle relaxation [50]. Oleuropein exerts potent antioxidant activities, such as inhibition of low density lipoproteins oxidation and free radical scavenging. In addition oleuropein modifies pathophysiological processes at the cellular level by inhibiting the production of superoxide anions, thromboxane and leukotriene B_4_, by neutrophils and by reducing platelet aggregation [47].

Wang *et al.* described the anti-atherosclerotic effect of oleuropein and its relationship with inflammatory response, using an experimentally rabbit model of atherosclerosis [51]. The administration of oleuropein was able to decrease serum levels of lipids and suppressed the development of atherosclerosis, downregulating the expression of TNF-α, which, in turn, decreased the expression of the monocyte chemotactic protein-1 and vascular cell adhesion molecule. Oleuropein, therefore, attenuates atherosclerosis via several mechanisms, including lowering lipids, inhibiting LDL oxidation, suppressing inflammatory factors and preventing macrophage activation [52,53].

### 2.3. Anti-Cancer and Anti-Angiogenic Effect

Several epidemiological studies have demonstrated that the incidence of some types of cancer in the Mediterranean basin is lower compared to other areas [54]; this favorable outcome has been ascribed to the Mediterranean diet. The Lyon Diet Heart Study was the first large randomized clinical trial demonstrating the beneficial effects of the Mediterranean diet in reducing mortality by 56% and in decreasing the cancer risk by 61% in a four-year follow up [55].

The Mediterranean diet has been inscribed by the United Nations Educational, Scientific and Cultural Organization (UNESCO) on the representative list of the intangible cultural heritage of humanity. Countries in the Mediterranean area are characterized by economic, cultural, religious and ethnical disparities. Moreover, agricultural production and the availability of fish and meat are different along the Mediterranean basin, profoundly affecting the local diet. Nonetheless, the so-called Mediterranean diet is characterized by consumption of large quantities of vegetables, legumes, fruits, whole grains and ﬁber-containing foods, ﬁsh, low-fat dairy, moderate wine intake, low red meat consumption and high intake of monounsaturated fatty acids derived from olive oil.

Compelling evidence has pointed to olive and olive oil consumption as an important factor for the beneficial effects on health of the Mediterranean diet [56]. Indeed, virgin olive oil consumption and the incidence of some forms of cancers, including colon, breast and skin cancer, are inversely correlated [57]. Several mechanisms have been proposed to account for the anti-tumor properties of the virgin olive oil [58,59]. Olive oil may act in reducing environmental and food carcinogens bioavailability. Most importantly, virgin olive oil may exert its antineoplastic function by protecting cells from oxidative stress. This is a consequence of its high content of oleic acid, which is less susceptible to oxidation than *n*-6 polyunsaturated fatty acids present in other edible oils (palm, peanut, soybean and sun ﬂower) and for the presence of a high content of antioxidant components (such as hydroxytyrosol and oleuropein), which are potent reactive oxygen species (ROS) scavengers. The beneficial effects of olive oil may in part be attributable to the ability of polyphenolic compounds to induce epigenetic modulation [60] and/or altered miRNA expression [61].

Amongst the minor antioxidants present in olive oil and olives, oleuropein has been indicated as the one responsible for the major anti-tumor activity [62]. Several lines of *in vitro* evidence have been collected demonstrating the antiproliferative and proapoptotic effects of oleuropein in different cancer cell lines [63] (Table 2). In addition, studies on experimental animals have determined that oleuropein treatment prevents development of skin [64], soft tissue [62] and breast cancer [65].

**Table 2:** Oleuropein-induced anti-tumor effects in different cancer cell lines.

Cell Line	Cancer Type	References
MCF-7	Breast adenocarcinoma	[62,65,66,67]
MDA	Breast adenocarcinoma	[68]
T-47D	Breast ductal carcinoma	[62]
HT 29	Colorectal adenocarcinoma	[69]
Caco-2	Colorectal adenocarcinoma	[70]
LoVo	Colorectal adenocarcinoma	[62]
TF 1	Erythroleukemia	[62]
LN 18	Glioblastoma	[62]
A549	Lung carcinoma	[71]
RPMI 7951	Melanoma	[62]
LNCaP and DU145	Prostate cancer	[72]
786-O	Renal cell adenocarcinoma	[62]
T-24	Urinary bladder carcinoma	[66]

Besides acting directly on tumor cells, oleuropein antitumor activity may be related to its anti-angiogenic function. Tumors contain several cell types that interact with each other and with the surrounding tissue, creating a complex interacting network within a permissive microenvironment. The stromal components support tumor growth and promote invasion through the stimulation of cancer cell proliferation, migration and invasion. Growth of the tumor mass creates a nutrient and oxygen-deprived environment, which induces the activation and proliferation of endothelial cells (EC) to sprout new vessels from pre-existing ones. This process, termed angiogenesis, is complex and tightly regulated, resulting from the balance between multiple angiogenic and anti-angiogenic factors from the tumor and the surrounding host cells.

In recent years, major efforts have focused on identifying and testing anti-angiogenic compounds as cancer therapeutics. Several anti-angiogenic agents are currently under investigation in clinical trials. Most of them are small molecule inhibitors targeting molecular mediators of angiogenesis and growth factor receptors, such as vascular endothelial growth factor receptor (VEGFR). Others are specific monoclonal antibodies, such as bevacizumab, which targets VEGF and has been approved by the Food and Drug Administration (FDA) for the treatment of several cancers, including metastatic colon cancer, pulmonary carcinoma, metastatic breast cancer, renal and ovarian cancer, and ramucirumab, a monoclonal antibody against VEGFR2, for metastatic gastric adenocarcinoma.

In this scenario, oleuropein has been described as an inhibitor of endothelial proliferation, and this use was patented [73]. In support of the patent application, Hamdi *et al.* provide evidence collected using both the CAM (chick chorioallantoic membrane) assay, traditionally used to assess the anti-angiogenic property of various compounds to study neonatal angiogenesis, and an “Adult Mouse Ear Model” to study the effect of oleuropein associated with wound healing. In both models, after oleuropein treatment, they observed a reduction of the number of blood vessels, indicating oleuropein as an anti-angiogenic factor.

Furthermore, Kimura *et al.* [64] administered oleuropein (10 and 25 mg/kg) orally twice a day before and after UVB irradiation, for 30 weeks. Their results suggest that the preventative effects of oleuropein on chronic UVB-induced skin damage and carcinogenesis and tumor growth may be due to the inhibition of the expression of VEGF, as well as of MMP-2, MMP-9 and MMP-13 through a reduction in COX-2 levels.

Moreover, Scoditti *et al.* [74] presented data on angiogenesis inhibition by oleuropein and its derivative hydroxytyrosol. They showed a significant inhibition of endothelial tube formation and migration, targeting endothelial cells, so providing more insights into the potential direct role of oleuropein in interfering with the multifaceted process of angiogenesis.

In this regard, Hamdi and Castellon [62] refer to oleuropein as a new class of anti-cancer compound, which targets multiple steps in cancer progression. As an antioxidant, it may protect cells from incurring genetic damage leading to oncogenesis. As an anti-angiogenic agent, it can prevent tumor progression. Finally, by directly inhibiting cancer cells, it can lead to tumor regression.

### 2.4. Hepatoprotective Effect

Olive oil may be helpful in reducing the progression of non-alcoholic fatty liver disease (NAFLD), a pathological condition in which fatty infiltration in the liver exceeds 5%–10% of its weight [75].

**Figure 1:**
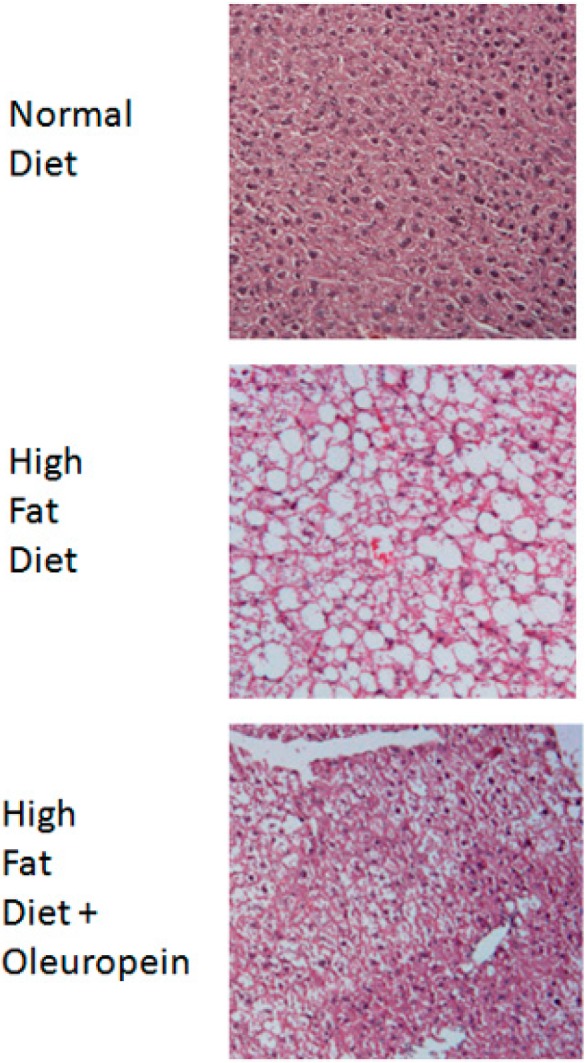
Oleuropein supplementation reduces hepatic lipid accumulation in high fat diet-treated mice. C57BL/6 mice were fed with a high fat diet (HFD) for eight weeks; then, animals were randomly divided into three groups of six mice each: the first received a normocaloric diet (ND), the second HFD, the third HFD supplemented with 3% oleuropein (HFD + Ole), for a further eight weeks. Mice were sacrificed, and histology was performed on sections of liver tissue, indicating reduced lipid deposition in the HFD + Ole group. Hematoxylin and eosin stain; original magnification: 40×. All experimental procedures conformed to protocols approved by the Institutional Animal Care and Use Committee (178/2013 B, on 16 July 2013) and were performed according to the Guidelines of the Italian National Institutes of Health.

Oleuropein administration has an hepatoprotective and therapeutic effects on carbon tetrachloride-induced liver damage in mice [76]. Moreover, a diet supplemented with oleuropein reduces induced hepatic steatosis [77] and progression to non-alcoholic steatohepatitis (NASH) [78] in mice fed with a high fat diet. Data collected in our laboratory confirmed that oleuropein supplementation is able to diminish lipid accumulation in the liver of mice fed with a high fat diet (Figure 1).

**Figure 2:**
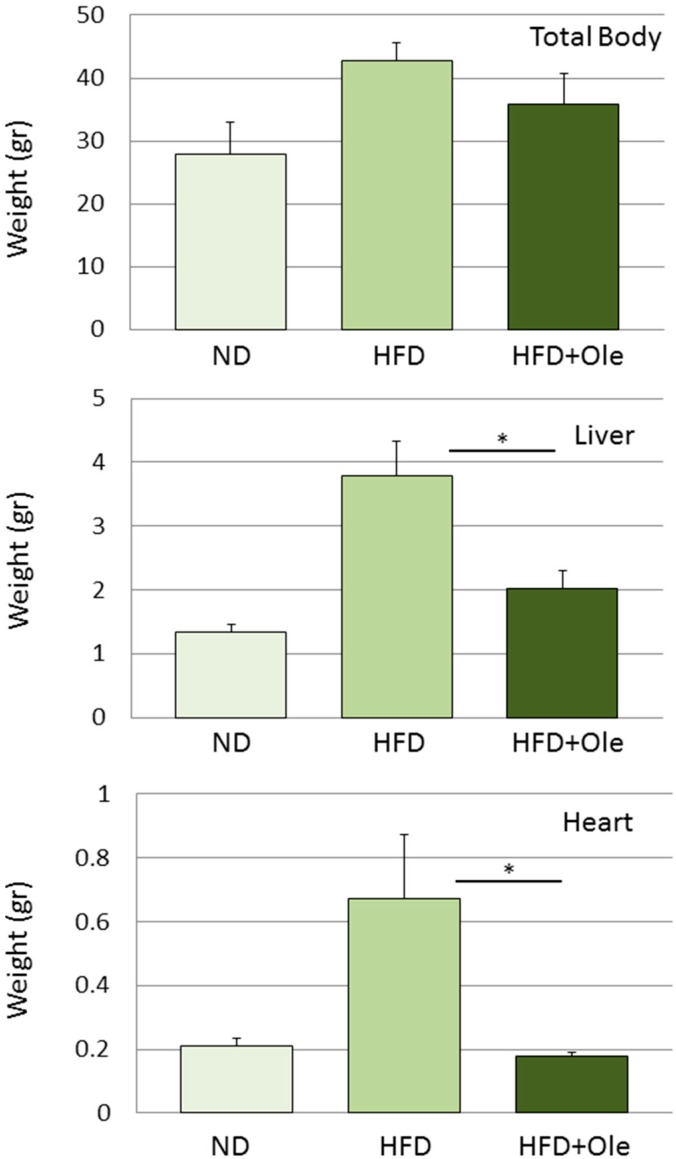
Oleuropein supplementation reduces body, liver and heart weights in high fat diet-treated mice. C57BL/6 mice were fed with a high fat diet (HFD) for eight weeks; then, animals were randomly divided into three groups: the first received a normocaloric diet (ND), the second high fat diet (HFD), the third HFD supplemented with 3% oleuropein (HFD + Ole) for further eight weeks. At the end of treatment, HFD + Ole mice had reduced weight gain (total body—25%, liver—50%, heart—70%) compared to HFD-fed mice. Data are expressed as the mean; error bars indicate the standard error; number of animals per group = 6. An asterisk denotes a statistically significant difference between HFD and HFD + Ole groups (*p* < 0.05 assessed with a two-tailed Student’s *t*-test for unpaired data).

Moreover, we observed also a reduction in the increment of body, liver and heart weights in mice fed with an oleuropein-enriched diet (Figure 2). Previous work performed in rats fed with oil leaf extract has suggested that liver weight reduction might be attributable to reduced collagen and fat deposition [79]. This effect of oleuropein has been associated with down-regulation of hepatic lipogenesis [80] and up-regulation of visceral fat thermogenesis [81].

### 2.5. Antimicrobial and Antiviral Effects

The presence of oleuropein in several parts of the plant (Table 1) confers to *Olea europaea* L. natural resistance to microbe attack [82]. Oleuropein exerts its antimicrobial activity against both Gram negative and positive bacteria, including *Lactobacillus plantarum*, *Bacillus cereus* and *Salmonella enteritidis* [83]. Moreover, *in vitro* studies revealed oleuropein antimycoplasmal activity, also against mycoplasma strains resistant to common antibiotic treatments [84].

For its antimicrobial properties, oleuropein can be used as a food additive [85] and for the treatment of human intestinal or respiratory tract infections [86]. The molecular mechanisms underlying oleuropein antimicrobial activity are still unclear [14].

Oleuropein also possesses a well-documented antiviral activity [14]. Its efficacy against hemorrhagic septicemia rhabdovirus (VHSV) [87], hepatitis B virus (HBV) [88] and human immunodeficiency virus (HIV) [89,90] was demonstrated. The beneficial effect of oleuropein against VHSV is exerted through a virucidal effect, reducing virus infectivity and avoiding cell-to-cell fusion of uninfected cells, probably acting on the virus envelope [87]. Oleuropein treatment efficiently blocks the secretion of hepatitis B surface antigen from infected HepG2 2.2.15 and reduces the viremia in duck infected with hepatitis B virus [88]. Oleuropein’s action against HIV has been correlated to its ability to bind and inhibit in a dose-dependent manner HIV-1 integrase activity. Oleuropein, therefore, represents a suitable molecular template that should facilitate the identification and design of innovative HIV-1 integrase inhibitors [90].

### 2.6. Neuroprotective Effect

Many neurodegenerative diseases, including Parkinson’s and Alzheimer’s, occur as a result of a progressive loss of the structure or function of neurons. Mitochondrial DNA damage and oxidative stress, both key factors in aging, contribute to the development and progression of degenerative diseases [91]. Some studies have been performed in order to evaluate the potential neuroprotection activity of oleuropein [47]. In particular, intra-peritoneal administration of oleuropein once a day for six months in aged rats improved the antioxidant enzyme activities in midbrains in comparison to the control group [92]. Furthermore, treated rats had more neurons in the substantia nigra, revealing that oleuropein protects against dopaminergic neuron loss. This result provides new hope in preventing or attenuating damage or loss of dopaminergic neurons associated with Parkinson’s disease.

Oleuropein was also indicated as a molecule with a therapeutic potential against Alzheimer’s disease. Oleuropein, in fact, has been described as a non-covalent binding molecule for amyloid-β (Aβ) 1–40 peptide [93]. The abnormal production of Aβ (1–40), due to the β- and γ-secretase cleavages of amyloid precursor protein and its aggregation, underline the deposition of the amyloid plaques, the hallmark of Alzheimer’s disease. Interestingly, mass spectrometry investigations revealed that the amino acids involved in oleuropein binding are crucial for the Aβ (1–40) peptide polymerization and fibril formation [94], indicating that oleuropein may exert a protective action counteracting the amyloid plaque generation and deposition. In addition, oleuropein is an inhibitor of Tau, a microtubule-associated protein known to aberrantly form amyloid-positive aggregates characteristic of Alzheimer’s disease [95]. A study on olive leaf extract containing oleuropein administrated intraperitoneally in adult male mice revealed an important effect on NGF and BDNF levels in olfactory bulbs and in brain, which are areas involved in neuritogenesis [96]. Moreover, oleuropein may be effective at protecting rat spinal cord from secondary injury [97].

Taken together, oleuropein can be ascribed to being one of the most important neuroprotective polyphenols.

## 3. Conclusions

Olive oil and olive leaf extract are renowned natural traditional remedies used for the treatment of different conditions, including dermatitis, wound healing and treatment of burns, stomach and intestinal pain, malaria-induced fever, different infections, alopecia, rheumatic pain, otitis, rickets, distortions, sciatica, hypertension, as a diuretic, as a laxative and as an aphrodisiac [60,98].

In light of the unique combination properties of oleuropein (Table 3), it looks like we should “go back to the future” and continue to exploit this key dietary component of the Mediterranean diet to promote human health.

**Table 3:** Biological activities and effect(s) of oleuropein.

Activity	Effect(s)	References
Antioxidant	Improvement of radical stability	[14,30]
	ROS scavenging effect	[30]
	Inhibition of oxidation of LDL	[36]
Anti-inflammatory	Inhibition of synthesis of pro-inflammatory cytokines	[39,47]
	Lipoxygenase inhibition	[38]
Anti-tumor	ROS scavenging effect	[56]
	Antiproliferative effect	[66,72]
	Apoptosis induction	[99]
	Anti-migration effect	[62,71]
	Angiogenesis inhibition	[73]
Hepatoprotective	Steatosis reduction	[77]
	Oxidative stress reduction	[76]
Antimicrobial	Bacterial cell membrane damage	[14]
Antiviral	Viral integrase inhibition	[89,90]
	Viral envelope interaction	[87]
Neuroprotective	Oxidative stress reduction	[47]
	Tau fibrillization inhibition	[95]

## References

[1] Ghanbari R., Anwar F., Alkharfy K.M., Gilani A.H., Saari N. (2012). Valuable nutrients and functional bioactives in different parts of olive (*Olea europaea* L.)-A review. Int. J. Mol. Sci..

[2] Visioli F., Galli C. (2002). Biological properties of olive oil phytochemicals. Crit. Rev. Food Sci. Nutr..

[3] Cicerale S., Conlan X.A., Sinclair A.J., Keast R.S. (2009). Chemistry and health of olive oil phenolics. Crit. Rev. Food Sci. Nutr..

[4] Cicerale S., Lucas L., Keast R. (2010). Biological activities of phenolic compounds present in virgin olive oil. Int. J. Mol. Sci..

[5] Vissers M.N., Zock P.L., Roodenburg A.J., Leenen R., Katan M.B. (2002). Olive oil phenols are absorbed in humans. J. Nutr..

[6] Shasha B., Leibowitz J. (1961). On the oleuropein, the bitter principle of olives. J. Org. Chem..

[7] Charoenprasert S., Mitchell A. (2012). Factors influencing phenolic compounds in table olives (*Olea europaea*). J. Agric. Food Chem..

[8] Goldsmith C.D., Stathopoulos C.E., Golding J.B., Roach P.D. (2014). Fate of the phenolic compounds during olive oil production with the traditional press method. Int. Food Res. J..

[9] Shasha B., Leibowitz J. (1959). Oleuropeic acid: A new compound from *Olea europaea*. Nature.

[10] Savournin C., Baghdikian B., Elias R., Dargouth-Kesraoui F., Boukef K., Balansard G. (2001). Rapid high-performance liquid chromatography analysis for the quantitative determination of oleuropein in *Olea europaea* leaves. J. Agric. Food Chem..

[11] Ansari M., Kazemipour M., Fathi S. (2011). Development of a simple green extraction procedure and HPLC method for determination of oleuropein in olive leaf extract applied to a multi-source comparative study. J. Iran. Chem. Soc..

[12] Tayoub G., Sulaiman H., Hassan A.H., Alorfi M. (2012). Determination of oleuropein in leaves and fruits of some Syrian olive varieties. Int. J. Med. Arom. Plants.

[13] Malik N.S.A., Bradford J.M. (2006). Changes in oleuropein levels during differentiation and development of ﬂoral buds in “Arbequina” olives. Sci. Hortic..

[14] Omar S.H. (2010). Oleuropein in olive and its pharmacological effects. Sci. Pharm..

[15] Altinyay C., Altun M.L. (2006). HPLC analysis of oleuropein in *Olea europaea* L. J. Fac. Pharm..

[16] Japón-Lujan R., Luque de Castro M.D. (2007). Small branches of olive tree: A source of biophenols complementary to olive leaves. J. Agric. Food Chem..

[17] Ortega-García F., Peragón J. (2010). HPLC analysis of oleuropein, hydroxytyrosol, and tyrosol in stems and roots of *Olea europaea* L. cv. Picual during ripening. J. Sci. Food Agric..

[18] Ranalli A., Contento S., Lucera L., di Febo M., Marchegiani D., di Fonzo V. (2006). Factors affecting the contents of iridoid oleuropein in olive leaves (*Olea europaea* L.). J. Agric. Food Chem..

[19] Bouaziz M., Jemai H., Khabou W., Sayadi S. (2010). Oil content, phenolic profiling and antioxidant potential of Tunisian olive drupes. J. Sci. Food Agric..

[20] Esti M., Cinquanta L., la Notte E. (1998). Phenolic compounds in different olive varieties. J. Agric. Food Chem..

[21] Zoidou E., Melliou E., Gikas E., Tsarbopoulos A., Magiatis P., Skaltsounis A.L. (2010). Identification of Throuba Thassos, a traditional Greek table olive variety, as a nutritional rich source of oleuropein. J. Agric. Food Chem..

[22] Perri E., Raffaelli A., Sindona G. (1999). Quantitation of oleuropein in virgin olive oil by ionspray mass spectrometry-selected reaction monitoring. J. Agric. Food Chem..

[23] Caponio F., Alloggio V., Gomes T. (1999). Phenolic compounds of virgin olive oil: Infuence of paste preparation techniques. Food Chem..

[24] Tuck K.L., Hayball P.J. (2002). Major phenolic compounds in olive oil: Metabolism and health effects. J. Nutr. Biochem..

[25] Tuberoso C.I., Kowalczyk A., Sarritzu E., Cabras P. (2007). Determination of antioxidant compounds and antioxidant activity in commercial oil seeds for food use. Food Chem..

[26] Visioli F., Galli C. (2000). Olive oil: More than just oleic acid. Am. J. Clin. Nutr..

[27] Kanakis P., Termentzi A., Michel T., Gikas E., Halabalaki M., Skaltsounis A.L. (2013). From olive drupes to olive oil. An HPLC-orbitrap-based qualitative and quantitative exploration of olive key metabolites. Planta Med..

[28] Allouche N., Fki I., Sayadi S. (2004). Toward a high yield recovery of antioxidants and purified hydroxytyrosol from olive mill wastewaters. J. Agric. Food Chem.

[29] Paiva-Martins F., Gordon M.H. (2005). Interactions of ferric ions with olive oil phenolic compounds. J. Agric. Food Chem..

[30] Visioli F., Bellomo G., Galli C. (1998). Free radical-scavenging properties of olive oil polyphenols. Biochem. Biophys. Res. Commun..

[31] Visioli F., Galli C. (1994). Oleuropein protects low density lipoprotein from oxidation. Life Sci..

[32] Visioli F., Bellomo G., Montedoro G., Galli C. (1995). Low density lipoprotein oxidation is inhibited *in vitro* by olive oil constituents. Atherosclerosis.

[33] Coni E., di Benedetto R., di Pasquale M., Masella R., Modesti D., Mattei R., Carlini E.A. (2000). Protective effect of oleuropein, an olive oil biophenol, on low density lipoprotein oxidizability in rabbits. Lipids.

[34] Visioli F., Caruso D., Galli C., Viappiani S., Galli G., Sala A. (2000). Olive oils rich in natural catecholic phenols decrease isoprostane excretion in humans. Biochem. Biophys. Res. Commun..

[35] Aruoma O.I., Halliwell B. (1987). Action of hypochlorous acid on the antioxidant protective enzymes superoxide dismutase, catalase and glutathione peroxidase. Biochem. J..

[36] De la Puerta R., Martínez Domínguez M.E., Ruíz-Gutíerrez V., Flavill J.A., Hoult J.R. (2001). Effects of virgin olive oil phenolics on scavenging of reactive nitrogen species and upon nitrergic neurotransmission. Life Sci..

[37] Manna C., Migliardi V., Golino P., Scognamiglio A., Galletti P., Chiariello M., Zappia V. (2004). Oleuropein prevents oxidative myocardial injury induced by ischemia and reperfusion. J. Nutr. Biochem..

[38] De la Puerta R., Ruiz Gutierrez V., Hoult J.R. (1999). Inhibition of leukocyte 5-lipoxygenase by phenolics from virgin olive oil. Biochem. Pharmacol..

[39] Giamarellos-Bourboulis E.J., Geladopoulos T., Chrisofos M., Koutoukas P., Vassiliadis J., Alexandrou I., Tsaganos T., Sabracos L., Karagianni V., Pelekanou E. (2006). Oleuropein: A novel immunomodulator conferring prolonged survival in experimental sepsis by Pseudomonas aeruginosa. Shock.

[40] Impellizzeri D., Esposito E., Mazzon E., Paterniti I., di Paola R., Bramanti P., Morittu V.M., Procopio A., Britti D., Cuzzocrea S. (2011). The effects of oleuropein aglycone, an olive oil compound, in a mouse model of carrageenan-induced pleurisy. Clin. Nutr..

[41] Schnell L., Fearn S., Schwab M.E., Perry V.H., Anthony D.C. (1999). Cytokine-induced acute inflammation in the brain and spinal cord. J. Neuropathol. Exp. Neurol..

[42] Pineau I., Lacroix S. (2007). Proinflammatory cytokine synthesis in the injured mouse spinal cord: Multiphasic expression pattern and identification of the cell types involved. J. Comp. Neurol..

[43] Khalatbary A.R., Zarrinjoei G.R. (2012). Anti-inflammatory effect of oleuropein in experimental rat spinal cord trauma. Iran. Red Crescent Med. J..

[44] Miles E.A., Zoubouli P., Calder P.C. (2005). Differential anti-inflammatory effects of phenolic compounds from extra virgin olive oil identified in human whole blood cultures. Nutrition.

[45] Fitó M., Cladellas M., de la Torre R., Martí J., Muñoz D., Schröder H., Alcántara M., Pujadas-Bastardes M., Marrugat J., López-Sabater M.C. (2008). Anti-inflammatory effect of virgin olive oil in stable coronary disease patients: A randomized, crossover, controlled trial. Eur. J. Clin. Nutr..

[46] Puel C., Mathey J., Agalias A., Kati-Coulibaly S., Mardon J., Obled C., Davicco M.J., Lebecque P., Horcajada M.N., Skaltsounis A.L. (2006). Dose-response study of effect of oleuropein, an olive oil polyphenol, in an ovariectomy/inflammation experimental model of bone loss in the rat. Clin. Nutr..

[47] Omar S.H. (2010). Cardioprotective and neuroprotective roles of oleuropein in olive. Saudi Pharm. J..

[48] Bulotta S., Celano M., Lepore S.M., Montalcini T., Pujia A., Russo D. (2014). Beneficial effects of the olive oil phenolic components oleuropein and hydroxytyrosol: Focus on protection against cardiovascular and metabolic diseases. J. Transl. Med..

[49] Andreadou I., Iliodromitis E.K., Mikros E., Constantinou M., Agalias A., Magiatis P., Skaltsounis A.L., Kamber E., Tsantili-Kakoulidou A., Kremastinos D.T. (2006). The olive constituent oleuropein exhibits anti-ischemic, antioxidative, and hypolipidemic effects in anesthetized rabbits. J. Nutr..

[50] Rocha B.S., Gago B., Barbosa R.M., Laranjinha J. (2009). Dietary polyphenols generate nitric oxide from nitrite in the stomach and induce smooth muscle relaxation. Toxicology.

[51] Wang L., Geng C., Jiang L., Gong D., Liu D., Yoshimura H., Zhong L. (2008). The anti-atherosclerotic effect of olive leaf extract is related to suppressed inflammatory response in rabbits with experimental atherosclerosis. Eur. J. Nutr..

[52] Bogani P., Galli C., Villa M., Visioli F. (2007). Postprandial anti-inflammatory and antioxidant effects of extra virgin olive oil. Atherosclerosis.

[53] Covas M.I., de la Torre K., Farré-Albaladejo M., Kaikkonen J., Fitó M., López-Sabater C., Pujadas-Bastardes M.A., Joglar J., Weinbrenner T., Lamuela-Raventós R.M. (2006). Postprandial LDL phenolic content and LDL oxidation are modulated by olive oil phenolic compounds in humans. Free Radic. Biol. Med..

[54] Gotsis E., Anagnostis P., Mariolis A., Vlachou A., Katsiki N., Karagiannis A. (2014). Health benefits of the Mediterranean diet: An update of research over the last 5 years. Angiology.

[55] De Lorgeril M., Salen P., Martin J.L., Monjaud I., Boucher P., Mamelle N. (1998). Mediterranean dietary pattern in a randomized trial: Prolonged survival and possible reduced cancer rate. Arch. Intern. Med..

[56] Owen R.W., Haubner R., Würtele G., Hull E., Spiegelhalder B., Bartsch H. (2004). Olives and olive oil in cancer prevention. Eur. J. Cancer Prev..

[57] Psaltopoulou T., Kosti R.I., Haidopoulos D., Dimopoulos M., Panagiotakos D.B. (2011). Olive oil intake is inversely related to cancer prevalence: A systematic review and a meta-analysis of 13,800 patients and 23,340 controls in 19 observational studies. Lipids Health Dis..

[58] Nan J.N., Ververis K., Bollu S., Rodd A.L., Swarup O., Karagiannis T.C. (2014). Biological effects of the olive polyphenol, hydroxytyrosol: An extra view from genome-wide transcriptome analysis. Hell. J. Nucl. Med..

[59] Escrich E., Moral R., Grau L., Costa I., Solanas M. (2007). Molecular mechanisms of the effects of olive oil and other dietary lipids on cancer. Mol. Nutr. Food Res..

[60] Caramia G., Gori A., Valli E., Cerretani L. (2006). Virgin olive oil in preventive medicine: From legend to epigenetics. Eur. J. Lipid Sci. Technol..

[61] Tunca B., Tezcan G., Cecener G., Egeli U., Ak S., Malyer H., Tumen G., Bilir A. (2012). *Olea europaea* leaf extract alters microRNA expression in human glioblastoma cells. J. Cancer Res. Clin. Oncol..

[62] Hamdi H.K., Castellon R. (2005). Oleuropein, a non-toxic olive iridoid, is an anti-tumor agent and cytoskeleton disruptor. Biochem. Biophys. Res. Commun..

[63] Casaburi I., Puoci F., Chimento A., Sirianni R., Ruggiero C., Avena P., Pezzi V. (2013). Potential of olive oil phenols as chemopreventive and therapeutic agents against cancer: A review of *in vitro* studies. Mol. Nutr. Food Res..

[64] Kimura Y., Sumiyoshi M. (2009). Olive leaf extract and its main component oleuropein prevent chronic ultraviolet B radiation-induced skin damage and carcinogenesis in hairless mice. J. Nutr..

[65] Sepporta M.V., Fuccelli R., Rosignoli P., Ricci G., Servili M., Morozzi G., Fabiani R. (2014). Oleuropein inhibits tumour growth and metastases dissemination in ovariectomised nude mice with MCF-7 human breast tumour xenografts. J. Func. Foods.

[66] Goulas V., Exarchou V., Troganis A.N., Psomiadou E., Fotsis T., Briasoulis E., Gerothanassis I.P. (2009). Phytochemicals in olive-leaf extracts and their antiproliferative activity against cancer and endothelial cells. Mol. Nutr. Food Res..

[67] Han J., Talorete T.P., Yamada P., Isoda H. (2009). Anti-proliferative and apoptotic effects of oleuropein and hydroxytyrosol on human breast cancer MCF-7 cells. Cytotechnology.

[68] Hassan Z.K., Elamin M.H., Daghestani M.H., Omer S.A., Al-Olayan E.M., Elobeid M.A., Virk P., Mohammed O.B. (2012). Oleuropein induces anti-metastatic effects in breast cancer. Asian Pac. J. Cancer Prev..

[69] Cárdeno A., Sánchez-Hidalgo M., Cortes-Delgado A., Alarcón de la Lastra C. (2013). Mechanisms involved in the antiproliferative and proapoptotic effects of unsaponifiable fraction of extra virgin olive oil on HT-29 cancer cells. Nutr. Cancer.

[70] Corona G., Deiana M., Incani A., Vauzour D., Dessì M.A., Spencer J.P. (2007). Inhibition of p38/CREB phosphorylation and COX-2 expression by olive oil polyphenols underlies their anti-proliferative effects. Biochem. Biophys. Res. Commun..

[71] Mao W., Shi H., Chen X., Yin Y., Yang T., Ge M., Luo M., Chen D., Qian X. (2012). Anti-proliferation and migration effects of oleuropein on human A549 lung carcinoma cells. Lat. Am. J. Pharm..

[72] Acquaviva R., di Giacomo C., Sorrenti V., Galvano F., Santangelo R., Cardile V., Gangia S., D’Orazio N., Abraham N.G., Vanella L. (2012). Antiproliferative effect of oleuropein in prostate cell lines. Int. J. Oncol..

[73] Hamdi H.K., Tavis R., Castellon R. (2003). Methods for inhibiting angiogenesis. U.S. Patent.

[74] Scoditti E., Calabriso N., Massaro M., Pellegrino M., Storelli C., Martines G., de Caterina R., Carluccio M.A. (2012). Mediterranean diet polyphenols reduce inflammatory angiogenesis through MMP-9 and COX-2 inhibition in human vascular endothelial cells: A potentially protective mechanism in atherosclerotic vascular disease and cancer. Arch. Biochem. Biophys..

[75] Assy N., Nassar F., Nasser G., Grosovski M. (2009). Olive oil consumption and non-alcoholic fatty liver disease. World J. Gastroenterol..

[76] Domitrović R., Jakovac H., Marchesi V.V., Šain I., Romić Ž., Rahelić D. (2012). Preventive and therapeutic effects of oleuropein against carbon tetrachloride-induced liver damage in mice. Pharmacol. Res..

[77] Park S., Choi Y., Um S.J., Yoon S.K., Park T. (2011). Oleuropein attenuates hepatic steatosis induced by high-fat diet in mice. J. Hepatol..

[78] Kim S.W., Hur W., Li T.Z., Lee Y.K., Choi J.E., Hong S.W., Lyoo K.S., You C.R., Jung E.S., Jung C.K. (2014). Oleuropein prevents the progression of steatohepatitis to hepatic fibrosis induced by a high-fat diet in mice. Exp. Mol. Med..

[79] Poudyal H., Campbell F., Brown L. (2010). Olive leaf extract attenuates cardiac, hepatic, and metabolic changes in high carbohydrate-, high fat-fed rats. J. Nutr..

[80] Kim Y., Choi Y., Park T. (2010). Hepatoprotective effect of oleuropein in mice: Mechanisms uncovered by gene expression profiling. Biotechnol. J..

[81] Shen Y., Song S.J., Keum N., Park T. (2014). Olive leaf extract attenuates obesity in high-fat diet-fed mice by modulating the expression of molecules involved in adipogenesis and thermogenesis. Evid. Based Complement. Altern. Med..

[82] Fleming H.P., Walter W.M., Etchells J.L. (1973). Antimicrobial properties of oleuropein and products of its hydrolysis from green olives. Appl. Microbiol..

[83] Cicerale S., Lucas L.J., Keast R.S. (2012). Antimicrobial, antioxidant and anti-inflammatory phenolic activities in extra virgin olive oil. Curr. Opin. Biotechnol..

[84] Furneri P.M., Marino A., Saija A., Uccella N., Bisignano G. (2002). *In vitro* antimycoplasmal activity of oleuropein. Int. J. Antimicrob. Agents.

[85] Durlu-Ozkaya F., Özkaya M.T. (2011). Oleuropein using as an additive for feed and products used for humans. J. Food Process. Technol..

[86] Bisignano G., Tomaino A., Lo Cascio R., Crisafi G., Uccella N., Saija A. (1999). On the *in vitro* antimicrobial activity of oleuropein and hydroxytyrosol. J. Pharm. Pharmacol..

[87] Micol V., Caturla N., Pérez-Fons L., Más V., Pérez L., Estepa A. (2005). The olive leaf extract exhibits antiviral activity against viral haemorrhagic septicaemia rhabdovirus (VHSV). Antivir. Res..

[88] Zhao G., Yin Z., Dong J. (2009). Antiviral efficacy against hepatitis B virus replication of oleuropein isolated from *Jasminum officinale* L. var. grandiflorum. J. Ethnopharmacol..

[89] Lee-Huang S., Huang P.L., Zhang D., Lee J.W., Bao J., Sun Y., Chang Y.T., Zhang J. (2007). Discovery of small-molecule HIV-1 fusion and integrase inhibitors oleuropein and hydroxytyrosol: Part I. fusion [corrected] inhibition. Biochem. Biophys. Res. Commun..

[90] Lee-Huang S., Huang P.L., Zhang D., Lee J.W., Bao J., Sun Y., Chang Y.T., Zhang J. (2007). Discovery of small-molecule HIV-1 fusion and integrase inhibitors oleuropein and hydroxytyrosol: Part II. integrase inhibition. Biochem. Biophys. Res. Commun..

[91] Lin M.T., Beal M.F. (2006). Mitochondrial dysfunction and oxidative stress in neurodegenerative diseases. Nature.

[92] Sarbishegi M., Mehraein F., Soleimani M. (2014). Antioxidant role of oleuropein on midbrain and dopaminergic neurons of substantia nigra in aged rats. Iran. Biomed. J..

[93] Bazoti F.N., Bergquist J., Markides K.E., Tsarbopoulos A. (2006). Noncovalent interaction between amyloid-β-peptide (1–40) and oleuropein studied by electrospray ionization mass spectrometry. J. Am. Soc. Mass Spectrom..

[94] Bazoti F.N., Bergquist J., Markides K., Tsarbopoulos A. (2008). Localization of the noncovalent binding site between amyloid-β-peptide and oleuropein using electrospray ionization FT-ICR mass spectrometry. J. Am. Soc. Mass Spectrom..

[95] Daccache A., Lion C., Sibille N., Gerard M., Slomianny C., Lippens G., Cotelle P. (2011). Oleuropein and derivatives from olives as Tau aggregation inhibitors. Neurochem. Int..

[96] Carito V., Venditti A., Bianco A., Ceccanti M., Serrilli A.M., Chaldakov G., Tarani L., de Nicolò S., Fiore M. (2014). Effects of olive leaf polyphenols on male mouse brain NGF, BDNF and their receptors TrkA, TrkB and p75. Nat. Prod. Res..

[97] Khalatbary A.R., Ahmadvand H. (2012). Neuroprotective effect of oleuropein following spinal cord injury in rats. Neurol Res..

[98] Waterman E., Lockwood B. (2007). Active components and clinical applications of olive oil. Altern Med. Rev..

[99] Cárdeno A., Sánchez-Hidalgo M., Rosillo M.A., Alarcón de la Lastra C. (2013). Oleuropein, a secoiridoid derived from olive tree, inhibits the proliferation of human colorectal cancer cell through down-regulation of HIF-1α. Nutr. Cancer.

